# Rapid fingerprinting of bacterial species using nanocavities created on screen-printed electrodes modified by β-cyclodextrin†

**DOI:** 10.1039/d3sd00074e

**Published:** 2023-06-16

**Authors:** Niloofar Haghighian, Ritu Kataky

**Affiliations:** a Department of Chemistry, University of Durham Lower Mountjoy Durham DH1 3LE UK Ritu.Kataky@Durham.ac.uk

## Abstract

Rapid and precise identification of infectious microorganisms is important across a range of applications where microbial contamination can cause serious issues ranging from microbial resistance to corrosion. In this paper a screen-printed, polymeric β-cyclodextrin (β-CD) modified electrode, affording nanocavities for inclusion of the analytes, is shown as a disposable sensor capable of identifying bacteria by their metabolites. Three bacterial species were tested: two from the *Pseudomonas* genus, *Pseudomonas fluorescens* (*P. fluorescens*) and *Pseudomonas aeruginosa* (*P. aeruginosa*), and *Serratia marcescens* (*S. marcescens*), a member of the family, *Enterobacteriaceae*. On biofilm formation each species gave distinct, reproducible, redox fingerprints with a detection limit of 4 × 10^−8^ M. Square wave adsorptive stripping voltammetry (SWAdSV) was used for detection. Scanning electron microscopy (SEM) and cyclic voltammetry (CV) techniques were used to characterize the morphology and electrical conductivity of the modified electrode. In comparison to the bare screen-printed electrode, the modified electrode showed a considerably higher performance and offered an excellent sensitivity along with a relatively fast analysis time.

## Abstract 2

Rapid and precise identification of infectious microorganisms is important across a range of applications where microbial contamination can cause serious issues ranging from microbial resistance to corrosion.

## Graphical abstract

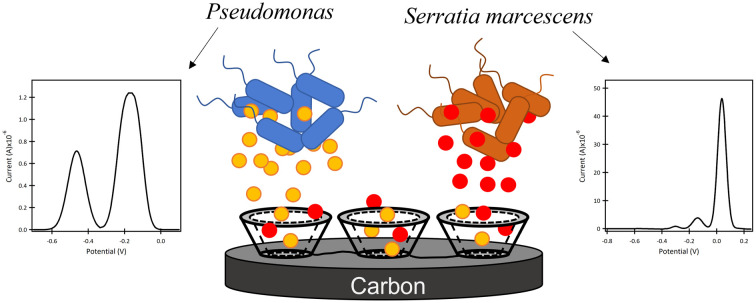

## Introduction

Label free and rapid identification of pathogenic bacteria, as opposed to detection of polymicrobial bacterial propagation, enables the identification and remediation of clinical diagnostics, food safety, and public health safety amongst others. Microbiological methods that are traditionally used, are time consuming. A rapid, on-site, affordable diagnostic capability is therefore, highly desirable.

In this work, fingerprint redox signatures of three diverse bacterial species have been studied, including two from the *Pseudomonas* genus, *P. fluorescens* and *P. aeruginosa*, and *S. marcescens*, a member of the family, *Enterobacteriaceae*. *P. fluorescens* and *P. aeruginosa* are both biofilm forming bacterial species of the *Pseudomonas* genus. They are both Gram-negative, rod-shaped, polar flagellated and aerobic. However, there is a key difference: *P. aeruginosa* is an opportunistic human pathogen which is virulent while *P. fluorescens* is a plant growth promoting bacterium. Several bacterial species, including *P. fluorescens* and *P. aeruginosa*, produce different variants of phenazines as secondary metabolites and quorum sensing molecules (Table 1).^1^ Both *P. fluorescens* and *P. aeruginosa* have the operons for the production of phenazine-1-carboxylic acid (PCA) from chorismates. However, only *P. aeruginosa* has diverse and specific enzymes required for the transformation of PCA to other phenazines such as phenazine-1-carboxamide (PCN), pyocyanin (5-*N*-methyl-1-hydroxyphenazine, PYO), and 1-hydroxyphenazine (1-OHPHZ). PYO production is associated with a high percentage of *P. aeruginosa* isolates and is considered to be the most potent virulence factor associated with the bacteria. These redox-active pigments control the redox status, gene expressions and metabolic flux and have been reported to influence antibiotic susceptibility.^2^

**Table 1:** List of phenazine metabolites present in *Pseudomonas* species, their redox mechanism and redox potential in aqueous solution^1,20,40^

Chemical name (abbreviation)	Redox couple involving two-electron transfer	*E* ^1/2^ (*vs.* Ag/AgCl) (mV), pH 7	*P. f*	*P. a*
Pyocyanin (PYO)	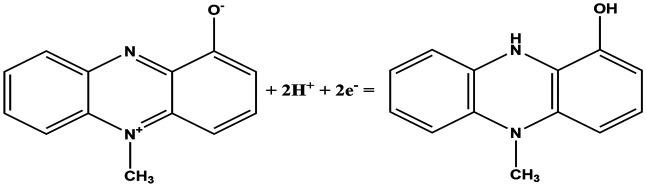	−245 (ref. 39)	✗	✓
Phenazine-1-carboxylate (PCA)	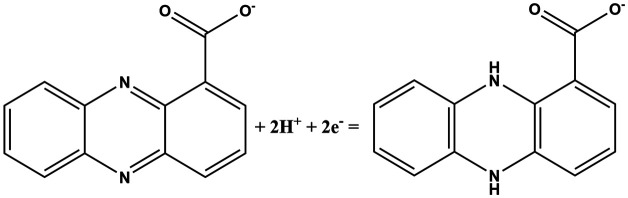	−321	✓	✓
1-Hydroxyphenazine (1-OHPHZ)	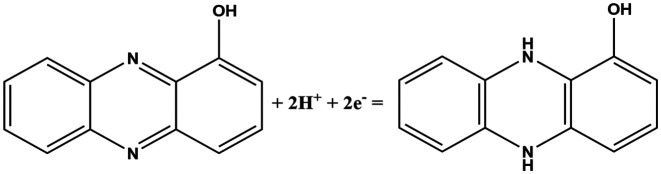	−379	✗	✓
Phenazine-1-carboxamide (PCN)	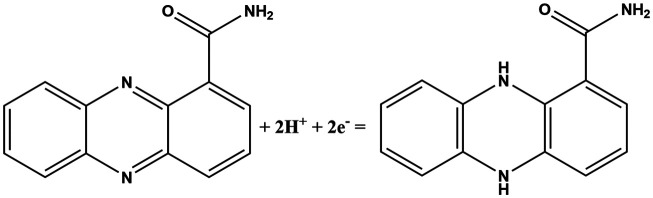	−345	✗	✓

**Scheme 1:**
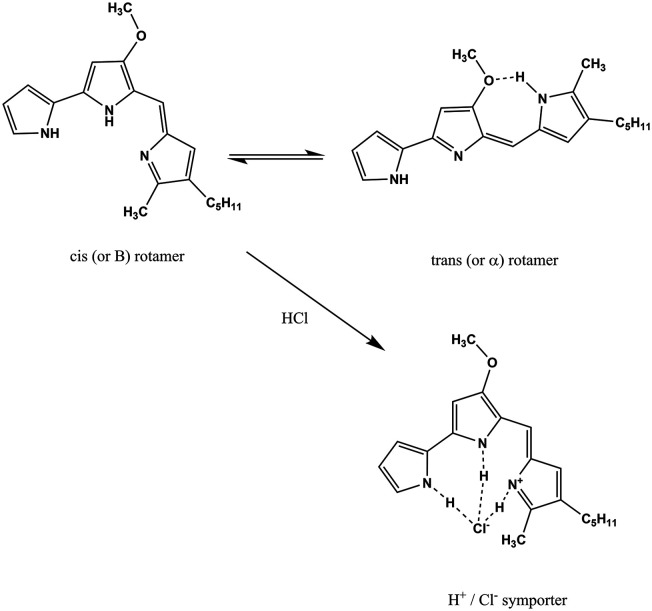
The structure of prodigiosin. Prodigiosin exists in solution as a mixture of *cis* (or β) and *trans* (or α). The balance between these forms is dependent on the pH of the solution as the *trans* form protonates more easily.^4^

*S. marcescens* is an opportunistic, Gram-negative pathogen, which is widespread in the environment and can cause hospital acquired infections such as urinary tract infections, respiratory tract infections and wound infections.^3^*Serratia* species are capable of producing a pigment, prodigiosin, as a secondary metabolite. Prodigiosin production is dependent on ambient conditions such as media composition, temperature, and pH. Structurally, it contains three pyrrolic rings, with a pyrrolyl dipyrromethene skeleton and a 4-methoxy, 2–2 bi pyrrole ring system (Scheme 1). The molecule has been extensively studied using spectroscopic methods. It can exist in two forms in solution as a mixture of cis (or β) and trans (or α) rotamers in a ratio that is dependent on the pH of the solution.^4^ In ethanol–water mixtures, prodigiosin has a p*K*_a_ value of 7.2.^5^ The molecule is reported to show two peaks in the visible part of the spectrum with maxima at 537 nm and 470 nm (2.31 and 2.64 eV, respectively). The lower energy peak dominates at acidic pH and the higher energy one at basic pH. Prodigiosin production is commonly estimated spectrophotometrically using the Haddix and Werner methods.^6^

Electrochemical sensing as a technique for pathogen identification *via* detection of redox-active metabolites on an electrode surface presents a straightforward electrochemical sensing approach. In a previous work, Bukelman and co-workers^7^ demonstrated that quorum sensing (QS) regulated virulence factor production can be analysed electrochemically, for the accurate and sensitive evaluation of QS activation and inhibition in wild-type bacteria. Buzid and co-workers^8^ have reported the use of unmodified boron doped diamond (BDD) electrodes without modification for the simultaneous determination of PYO, PQS and HHQ in a mixed solution to analyse supernatant extracts from *P. aeruginosa* wild-type strains. This work was reported as an improvement in the limits of detection reported in previous works using BDD thin film electrodes where only PQS was measured.^9,10^ Other reports include the use of biosensing assays.^11–14^

PYO has been detected using SWAdSV, using a hanging mercury drop electrode, and differential pulse voltammetry (DPV), using graphite rods and disposable screen-printed electrodes by square wave voltammetry (SWV).^7,15–19^ Sensors suitable for integration into bandages and nanofluidic platforms, based on electrochemical detection have also been reported.^20–22^ For the detection of multiple phenazines such as PQS and PYQ, conductive polymer film modified glassy carbon electrodes^23^ and preconcentration techniques have been reported.^24^ An interesting paper reports an electrochemical camera chip capable of simultaneous spatial imaging of multiple redox-active phenazine metabolites produced by *P. aeruginosa*. PA14 colony biofilms on the colonies are supported by agar-soaked membranes placed directly on top of the chip.^25^ In a very recent publication, Oziat and co-workers used unmodified glassy carbon electrodes to differentiate between *P. aeruginosa* strains and its isogenic mutants using square wave voltammetry. They observed distinctive redox signals showing PYO and *Pseudomonas* quinolone signals.^26^ The capability of differentiating bacterial species directly, by the identification of specific metabolites that can act as a ‘fingerprint’ for the species, would provide a useful platform for monitoring bacterial activity.

In this paper, β-CD electropolymerized on the surface of a screen-printed carbon electrode is demonstrated as a sensor format capable of ‘fingerprinting’ redox-active bacteria. Cyclodextrins have toroidal hydrophobic cavities with a hydrophilic exterior and have been widely used for molecular recognition, due to their natural size and charge selective cavity.^27–31^ The CD cavities are known to provide large, catalytic enhancement of reactions when the geometry of the substrate–CD complex is optimal.^30^ β-cyclodextrins, used in this work, consist of seven glucose units. The structure of the β-CD (height of 0.79 ± 0.01 nm, exterior diameter 1.54 ± 0.04) enables the incorporation of lipophilic structures with appropriate sizes into its cavity.^27,32^ The planar structure of phenazines with a heterocyclic pyrazine core and a fully conjugated aromatic π-system, and the hydroxyl and carboxylate moieties in the phenazines in this study point to partial inclusion into the hydrophobic cavity with hydrogen bonding. Similarly, π-rich prodigiosin is also known to form partial inclusion complexes with β-CD, and hydrogen bonding interactions.^33^

In this paper, using electrochemical polymerization, a β-CD polymer was used to modify disposable SPCE. This modification combined with optimized adsorptive stripping voltammetry techniques enabled the identification of distinctive fingerprints for *P. fluorescens*, *P. aeruginosa* and *S. marcescens* species.

## Experimental method

### Chemical and instrument

1-OHPHZ (purity of 98%), PYO (purity of 98%), and β-CD powder were purchased from Sigma-Aldrich. Phenazine-1-carboxylic acid was provided by Apollo Scientific. 1-OHPHZ and PYO stock solution (4.0 × 10^−4^ μM) were prepared using ethanol–phosphate buffer solution (PBS pH 7, 1.01 g cm^−3^) (1 : 10) as solvent. PCA stock solution (4.0 × 10^−4^ μM) was prepared in PBS pH 7. Electrochemical measurements were performed using a single screen-printed electrode (Micrux Technologies, Gijón, Spain (S1PE)), consisting of a working electrode (carbon, diameter of 3 mm), carbon-based counter electrode and silver reference electrode. CV and SWAdSV measurements were performed with a Gamry PE-1000 potentiostat. The SWAdSV technique was applied under optimum conditions such as accumulation time (*t*_acc_) of 120 s, frequency (*f*) of 25 Hz, pulse amplitude (*E*_sw_) of 25 mV, step potential (Δ*E*_S_) of 2 mV and deposition potential of −0.8 V at β-CD modified screen-printed electrode. All measurements were made at pH 7.2 and ambient temperature. The surface morphology of the polymers was determined using scanning electron microscopy (Zeiss Sigma 300 VP).

### Bacterial culture

*P. fluorescens*, *P. aeruginosa* and *S. marcescens* has grown overnight at 37 °C with continuous shaking in 5 ml of LB growth media. Glucose (20 g L^−1^) was supplied as an electron donor.

### Preparation of modified β-CD/SPCE

The SPCE was then modified by continuous potential cycling from −2 to 2 V at a sweep rate of 20 mV s^−1^ for 10 cycles, in a solution containing 0.01 M β-CD in PBS pH 7.^34^ The cyclic voltammograms obtained by the β-CD electro-polymerization process on SPCE are shown in Fig. S1.† After the electrodeposition, the electrode was carefully washed with deionized water to remove adsorbed materials on the surface and then dried at a room temperature for further use. All information regarding the electrode characterization can be found in the ESI.†

## Result and discussion

### *Pseudomonas* species

#### The electrochemical behaviour of 1-OHPHZ, PCA and PYO (phenazine metabolites) on β-CD/SPCE

**Fig. 1:**
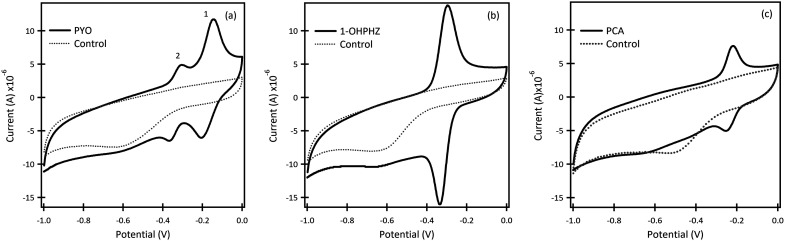
Cyclic voltammetry graphs of β-CD/SPCE in 50 μM (a) PYO, (b) 1-OHPHZ and (c) PCA in PBS at pH 7. Scan rate: 100 mV s^−1^. The dotted lines represent the blank which is PBS at pH 7.

Prior to detecting phenazines from *P. fluorescens* and *P. aeruginosa* biofilms, individual phenazines were monitored in PBS pH 7 on β-CD/SPCE for characterising their redox potentials. All three electrodes modified under the same conditions showed marginally different background plots. This is likely to be due to the electropolymerisation process resulting in somewhat varying structures, as they are not batch produced. However, background subtraction circumvents this issue. Three endogenous phenazines; PYO, PCN and 1-OHPHZ, were characterised individually, (Fig. 1a–c). The CV plots of 12.5 μM PYO, PCN and 1-OHPHZ in PBS, exhibited anodic (oxidation) and cathodic (reduction) peaks for PYO. The oxidation peaks occur at two distinct potentials around (*E*^1^_ox_ = −0.144 V, *E*^2^_ox_ = −0.312 V). Similarly, the reduction peaks occur at around (*E*^1^_re_ = −0.206 V, *E*^2^_re_ = −0.366 V).^21,35^ In contrast, only one well-defined redox peak for PCA and 1-OHPHZ was observed at (*E*^PCA^_ox_ = −0.218 V, *E*^PCA^_re_ = −0.248) and (*E*^1-OHPHZ^_ox_ = −0.298 V *E*^1-OHPHZ^_re_ = −0.336 V), respectively. The small value of the peak-to-peak separation (Δ*E*_P_ ≪ 0.06 V) for all three molecules suggest that the electron transfer process is likely to be quasi-reversible. Analysis of scan rate dependency showed that both cathodic and anodic peaks currents increased linearly for all three phenazine compounds with the *ν* as expected for the redox reaction of surface-confined molecules (Fig. S4a and b†).^35^

Phenazines are ionizable species, their p*K*_a_'s being highly dependent on the pH of the background solutions. Size, charge and shape selectivity influence the formation of inclusion complexes. In general, uncharged, neutral species are more likely to form inclusion complexes with the hydrophobic cavities of cyclodextrins. A detailed discussion of speciation and binding properties of phenazines with CD cavities is beyond the scope of this paper. Readers can access excellent references on the topic.^36^

**Fig. 2:**
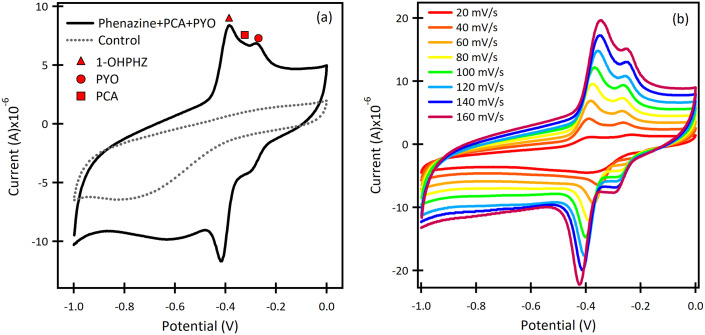
Cyclic voltammetry graphs of β-CD/SPCE (a) in mixtures of 12.5 μM 1-OHPHZ, PYO and PCA in PBS pH 7 (solid line) and control (dotted line), and (b) at different scan rates from 20 mV s^−1^ to 160 mV s^−1^ in 12.5 μM (1-OHPHZ + PYO + PCA).

The β-CD/SPCE tested in a ternary solution mixture of 1-OHPHZ, PCA and PYO in PBS pH 7 is shown in Fig. 2a. The CV graph exhibited well-defined redox peaks for 1-OHPHZ and PYO at −0.384 V and −0.278 V respectively, whereas a small, partially overlapped peak for PCA was observed in the voltammogram. To get information on the electrochemical reaction mechanism, the effect of scan rate on the peak current and potential were evaluated for a 12.5 μM ternary solution mixture of 1-OHPHZ, PCA and PYO in PBS pH 7 at the β-CD/SPCE. As shown in Fig. 2b, the anodic and cathodic peak current increased with the scan rate in the range 20 mV s^−1^ to 160 mV s^−1^. In the ternary mixtures, once again both cathodic and anodic peak currents increase linearly for all three phenazine compounds with the scan rate, confirming the redox reactions of surface-confined molecules.

#### SWAdSV of phenazine metabolites in *Pseudomonas* species

Following initial characterization using CV, SWAdSV was used as the preferred analytical technique for faster response times and higher sensitivity (Fig. S5†). The analyte was allowed to preconcentrate on the nanocavities of the β-CD at −0.8 V followed by cathodic stripping. The optimum conditions are PBS pH 7, accumulation time (*t*_acc_) of 120 s, frequency (*f*) of 25 Hz, pulse amplitude (*E*_sw_) of 25 mV, step potential (Δ*E*_S_) of 2 mV and deposition potential of −0.8 V at modified screen-printed β-CD. SWAdSV shows peak current 5 times larger than that of DPV (Fig. S5a–c†), confirming the inclusion of the phenazine metabolites in the CD cavity followed by stripping, enhanced resolution and sensitivity.

**Fig. 3:**
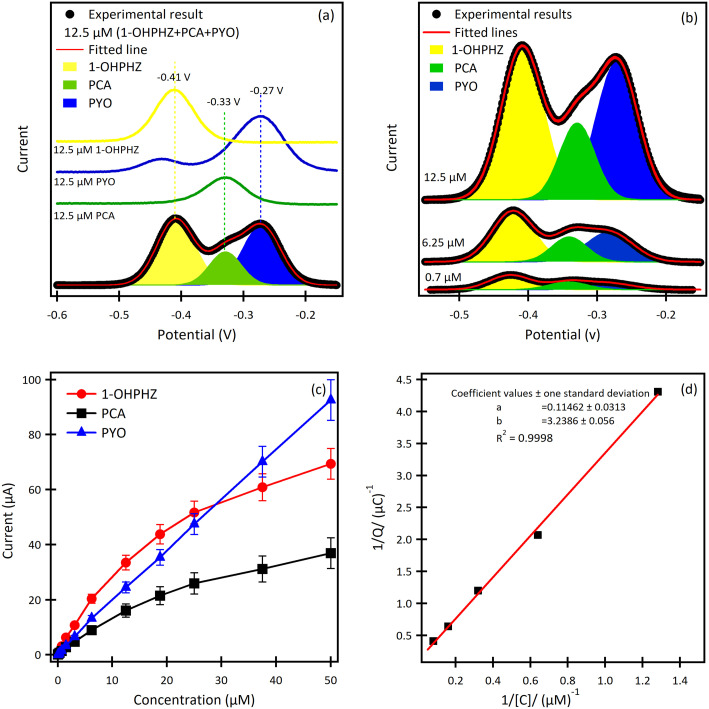
SWAdSV curves of (a) the ternary mixture of 12.5 μM 1-OHPHZ, PYO and PCA in PBS on β-CD/SPCE. 1-OHPHZ (yellow peaks), PCA (green peaks) and PYO (blue peaks) in both mixed and individual curves. (b) SWAdSV curves of the β-CD modified electrode for different concentrations of mixed 1-OHPHZ, PYO and PCA. (c) Calibration curves for 1-OHPHZ, PYO and PCA separately. (d) 1/*Q versus* 1/*C* Langmuir plot for of 1-OHPHZ on modified electrode.

The result for a ternary solution mixture of 12.5 μM 1-OHPHZ, PCA and PYO in PBS at β-CD/SPCE (Fig. 3a) showed peaks that could be deconvoluted to the three peaks corresponding to 1-OHPHZ, PYO and PCA individually, at −0.41 V, −0.33 V and −0.27 V (*vs.* Ag pseudo reference electrode), respectively. These three peaks all shifted 0.1 V toward the negative potential. Deconvolution of the net SWAdSV peaks (Fig. S6†) showed a forward (*I*_fwd_), a backward (*I*_back_), and a net (*I*_fwd_ − *I*_back_) voltammogram characteristic of a quasi-reversible oxidation process.

Comparison of SWASV using the same solution on bare SPCE (Table 2) under the same experimental conditions reveal a significant enhancement of signal obtained using β-CD/SPC, due to the pre-concentration of the analytes entrapped in the cavity of the β-CD in proxiomparison of the SWAdSV responsemity with the electrode surface under optimized conditions. The co-detection of 1-OHPHZ, PCA and PYO with increasing concentration from 0.08–50 μM were recorded by SWAdSV on β-CD modified SPCE (Fig. S7†). The electrochemical signals gradually increased as the concentration of 1-OHPHZ, PCA and PYO increased in the mixture. Fig. 3b depicts cathodic stripping voltammetric peaks at concentration of 0.7, 6.25 and 12.5 μM of the mixed molecules. Fig. 3c shows the corresponding calibration graphs for each molecule. The calibration graph is linear over the entire range of 0.08–50 μM for PYO, while that for PCA and 1-OHPHZ are linear over the range of 0.08–1.5 μM. The limit of detection (LOD) and limit of quantification (LOQ) of 10^−8^ M and 10^−7^ M were calculated for all three phenazines, respectively (Fig. S8†). This value enables sensitive detection of phenazines, enabling early detection of biofilms by quorum sensing production of phenazine released by colonising *P. aeruginosa* and *P. fluorescens*. To investigate the adsorption of phenazine metabolites on the modified electrode, these experimental data were fitted to the Langmuir isotherm adsorption model:^37^
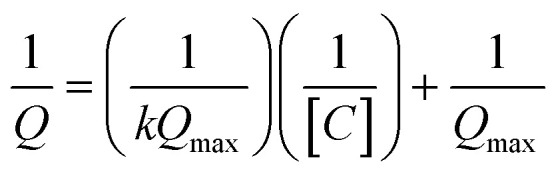
where *C* is the bulk concentration of phenazine, *Q* is the voltammetry measured charge transferred at *C*, *k* is the equilibrium constant for adsorption and *Q*_max_ is the maximum charge transfer at the saturated surface with phenazine. The plot of 1/*Q versus* 1/*C* of 1-OHPHZ is linear (see Fig. 3d) with a correlation coefficient of 0.998 for the β-CD modified electrode. Linear regression analysis of the data yielded *Q*_max_ = 8.7 μC from the slope and *k* = 0.035 μM^−1^. This value is larger in comparison to the un-modified electrode, due to the much-increased adsorption (Fig. S9†).

**Table 2:** Comparison of the SWAdSV response of 12.5 μM mixed 1-OHPHZ, PYO and PCA on bare and β-CD modified SPCE in PBS pH 7

	1-OHPHZ		PCA		PYO	
	Bare SPEC	β-CD/SPCE	Bare SPEC	β-CD/SPCE	Bare SPEC	β-CD/SPCE
*Q*_net_ (μC)	0.89	2.37	0.519	1.04	1.17	2.07
*I*_net_ (μA)	13.5	31.8	8.54	16.1	17.5	28.3
*E*_net_ (V)	−0.348	−0.408	−0.280	−0.328	−0.219	−0.269
	±0.001	±0.001	±0.01	±0.01	±0.001	±0.001

#### Electrochemical detection of the bacterial cultures, *Pseudomonas*

**Fig. 4:**
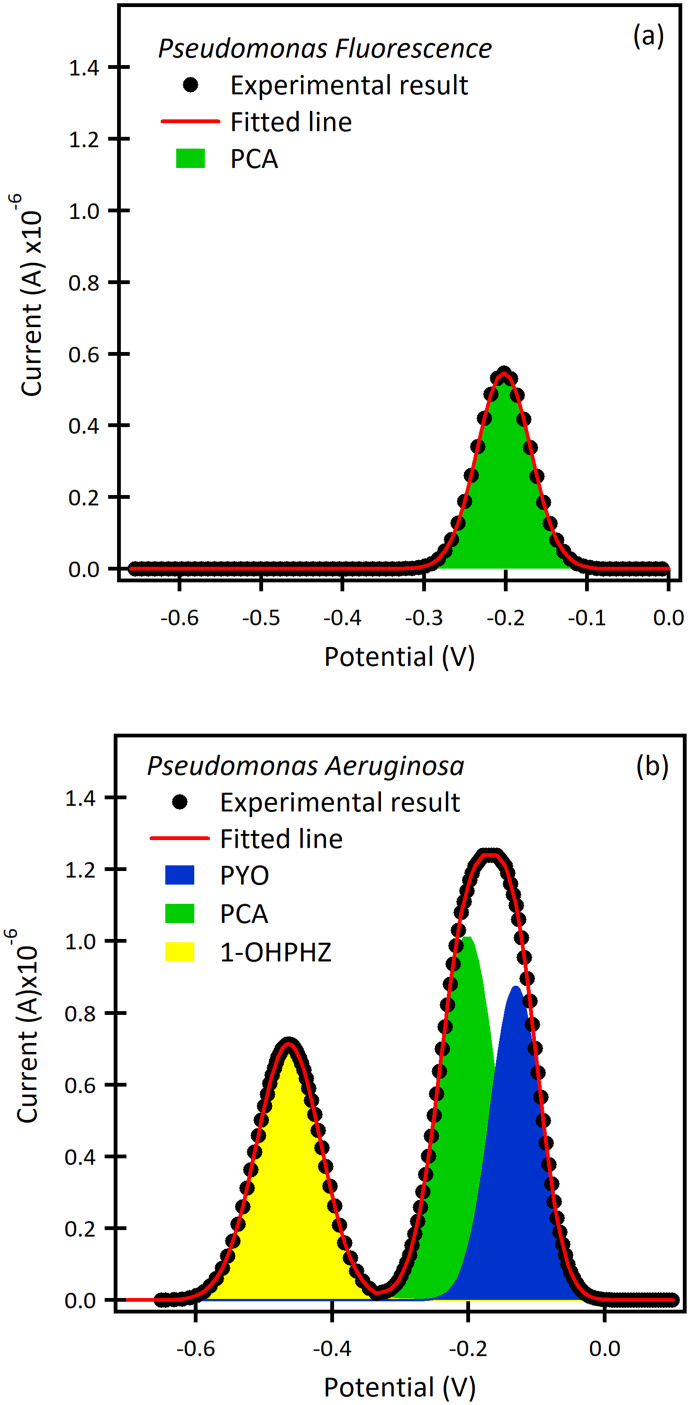
SWAdSV curves of the β-CD modified electrode after 72 h in (a) *P. fluorescens*, and (b) *P. aeruginosa* cultures. There is only one visible peak observed for *P. aeruginosa* at −0.20 V corresponding to the potential of PCA (green peak). *P. aeruginosa* shows multiple peaks at −0.46 V, −0.20 V and −0.13 V; it can be concluded that the peaks are from 1-OHPHZ (yellow peak), PCA (green peak) and PYO (blue peak), respectively.

The proposed method was applied for electrochemical detection of phenazine metabolites from *P. fluorescens* and *P. aeruginosa* in LB growth media at pH 7. Bacterial biofilms were allowed to grow on the β-CD modified SPCEs. SWAdSV was conducted at different time points during the biofilm formation, (Fig. S10†) which showed the SWSV response during growth. Fig. 4a and b show the SWAdSVs of *P. fluorescens* and *P. aeruginosa* in LB growth media after 3 days, respectively. There is only one visible peak observed for *P. fluorescens* at −0.20 V corresponding to the potential of PCA. According to the calibration curve, the estimated concentration of the PCA was calculated as 0.1 μM after 3 days. However, *P. aeruginosa* shows multiple peaks at −0.46 V, −0.20 V and −0.13 V. By comparison with the calibration curves, it can be concluded that the peaks are from 1-OHPHZ, PCA and PYO calculated concentrations of 0.2 μM, 0.5 μM and 0.6 μM, respectively. Phenazine is pH-sensitive so there is the possibility that the pH was slightly basic thus shifting the potential. These results agreed well with the previous findings that *P. aeruginosa* can secrete multiple phenazines, including PYO, PCA, PCN and 1-OHPHZ.^31,21^

#### S. marcescens

**Fig. 5:**
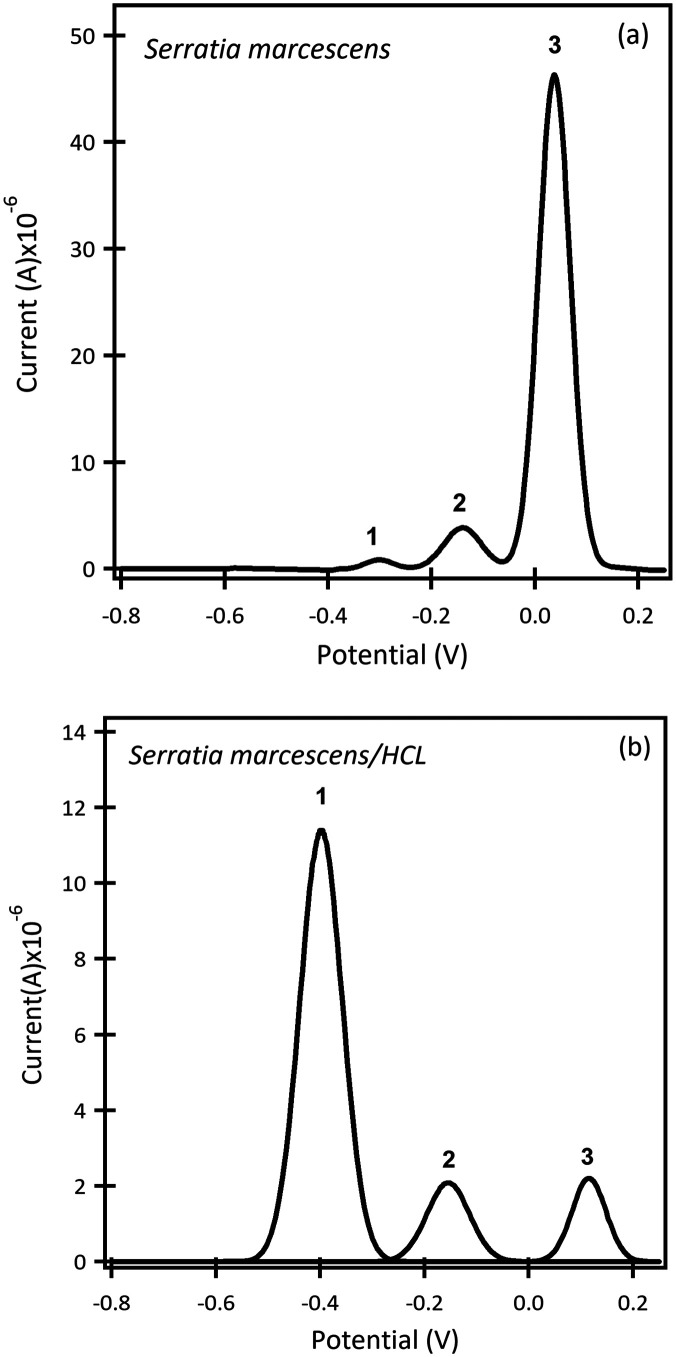
SWAdSV curves of the β-CD modified electrode after 4 days in (a) *S. marcescens* pH 7, and (b) after adding HCl to change the pH level to 3. The results in pH 7 revealed three peaks (*E*^1^ = −0.30 V, *E*^2^ = −0.14 V, *E*^3^ = 0.03 V) *vs.* Ag pseudo reference electrode; the peaks potential shifted to (*E*^1^ = −0.39 V, *E*^2^ = −0.15 V, *E*^3^ = 0.11 V) *vs.* Ag pseudo reference electrode in acidic conditions.

SWAdSV curves of *S. marcescens* in LB growth media after 3 days (Fig. 5a) revealed three peaks (*E*^1^ = −0.30 V, *E*^2^ = −0.14 V, *E*^3^ = 0.03 *vs.* Ag pseudo reference electrode). The three peak current intensities were (*i*^1^_p_ = 0.85 μA, *i*^2^_p_ = 3.6 μA and *i*^3^_p_ = 46 μA). In acidic conditions, (Fig. 5b) the peak potentials shifted to *E*^1^ = −0.39 V, *E*^2^ = −0.15 V, *E*^3^ = 0.11 V (*vs.* Ag pseudo reference electrode) with a change in peak current intensities (*i*^1^_p_ = 11.4 μA, *i*^2^_p_ = 2 μA and *i*^3^_p_ = 2.20 μA). In acidic media, the first peak was the most significant compared to the basic media where the third peak was predominant. There are very few reports of electrochemical measurements of prodigiosin. Melvin and co-workers performed CVs of pure acetonitrile using a three-electrode cell consisting of a glassy carbon working electrode, a Pt spiral counter-electrode, and a silver wire pseudo reference electrode.^38^ They reported three peaks at *E*^1^ = 0.44 V, *E*^2^ = 0.89 V and *E*^3^ = 1.54 V *vs.* SCE with the second peak showing a shoulder at 1.06 V. Under acidic conditions to generate the protonated species, a shift of *E*^2^ (0.62 V) and a slight decrease in *i*_p_ was reported. Although our measurements are not directly comparable, we observed three distinct redox peaks with an anodic shift in *E*^3^ accompanied by a decrease in current intensity. These results are consistent with the generation of the conjugate acid, which, as a positively charged species, is oxidized at a higher potential than the corresponding free base. Measurements at both neutral and acidic conditions are important because this opportunistic pathogen can cause infections in a wide range of health care associated applications including surgical cleaning solutions, disinfectant solutions, and digestive surgery amongst others. These microbes can also increase and decrease pH in soils, plants and other media.

## Conclusion

In conclusion, the results indicate that β-CD modified SPCE produced an excellent resolution of the redox peaks produced by quorum sensing molecules and metabolites of bacterial biofilms compared with the unmodified screen-printed electrode. This effect was produced by the catalytic enhancement effects of inclusion complexes of the complex between β-CD and quorum sensing molecules. The proposed method can deconvolute redox peaks of metabolites from different bacterial species, thus offering a simple method for identifying and fingerprinting bacterial species, which may have diagnostic and therapeutic applications.

## Conflicts of interest

There are no conflicts to declare.

## Supplementary Material

SD-002-D3SD00074E-s001
