# Supplementary material for: Rapid fingerprinting of bacterial species using nanocavities created on screen-printed electrodes modified by β-cyclodextrin
Source: Sens Diagn. 2023 Jun 16;2(5):1228–35. doi: 10.1039/d3sd00074e (PMC10501327; doi:10.1039/d3sd00074e)
Supplement: SD-002-D3SD00074E-s001 [file SD-002-D3SD00074E-s001.pdf]

Electronic Supplementary Material (ESI) for Sensors & Diagnostics.
This journal is © The Royal Society of Chemistry 2023

Supporting information

EXPERIMENTAL METHOD

Preparation of modified β-CD screen-printed carbon electrode

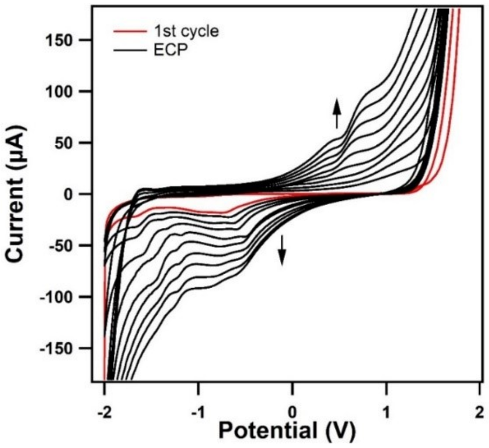

Figure S1: cyclic voltammograms obtained during the electro-polymerization of BCD (0.01 M) in PBS (pH7). CV was performed between -2 and 2 V (VS. Ag pseudo reference electrode).

Characterization of the electrode. The electrochemical characterization of the bare and β-CD/SPCE was performed by CV and EIS using a 5.0 mM [Fe (CN) 6]$^{-3/-4}$ in PBS. The CV responses bare and modified SPCEs in the potential range from -0.7 to +1 V at a scan rate of 100 mV/s. The presence of the polymer layer affects the electron transfer rate at the electrode surface, leading to changes in the current density and potential range of the redox reaction. The peak current (I_{pa}) at the modified SPCE in (Figure S2a) is much larger (14 μA) than that at the bare SPCE. This demonstrates that the deposition of Pβ-CD has substantially enhanced the electron transfer rate of SPCE. It is also evident that in the case of β-CD/SPCE, the peak-to-peak separation (ΔE_p) decreased from 0.67 V to 0.38 V compared to the case of the bare SPCE. Considering the small ΔE_p value and large current response of β-CD/SPCE, the electron transfer process is likely quick and quasi-reversible [1, 2].

Electrochemical impedance spectroscopy (EIS) measurements in a frequency range of 100 KHz to 0.1Hz were used to characterize interface properties of the bare and β-CD/SPCE. The Nyquist plot of electrochemical impedance spectra for bare and β-CD modifies SPCE, and Randle's equivalent circuit model were used to fit the experimental data over the whole frequency range are shown in (Figure S2b). This circuit includes charge transfer resistance (R_{ct}), diffusion impedance (W), solution resistance (Rs), and constant phase element (CPE) corresponding to the double-layer capacitance [3]. W is the Warburg impedance, which is related to the diffusion of the reactive species at the surface of the electrodes and is illustrated by the straight line in the Nyquist plots. At high frequency to medium frequency the charge transfer kinetics of the redox species [Fe (CN) 6]$^{-3/-4}$ at electrode/electrolyte interface (R_{ct}) is represented by

the diameter of the semicircle. The smaller R_{ct}, the faster the electron transfer rate, indicating better electrochemical activity of the modified electrode due to the increased surface area of the electrode.

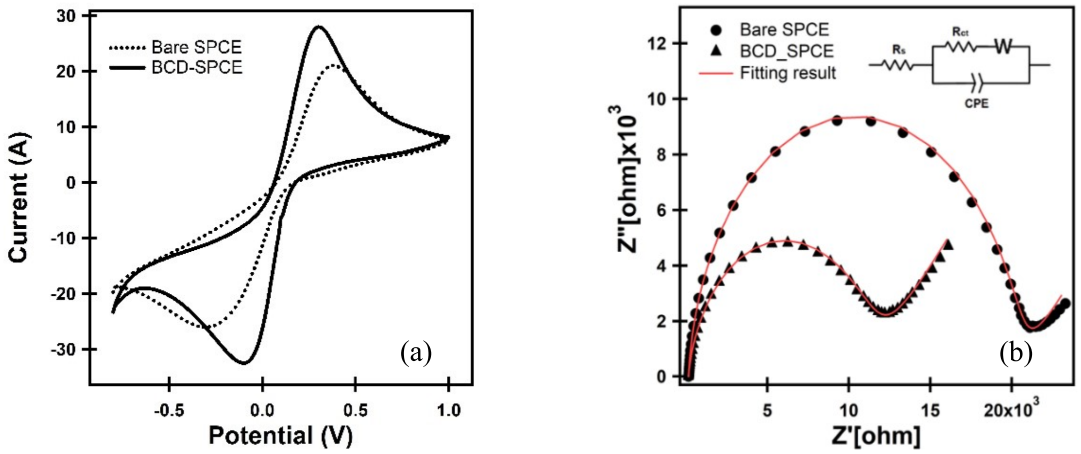

Figure S2. (a) CV plot and (b) Nyquist diagram of electrochemical impedance spectra for bare SPCE and β-CD modifies SPCE in 5.0 mM [Fe (CN)6]-3/-4. Randles- circuit used for fitting the experimental results (Inset of (b)), where Rs is solution resistance, CPE is constant phase element, R_{ct} is the charge transfer and W is diffusion hindered impedance.

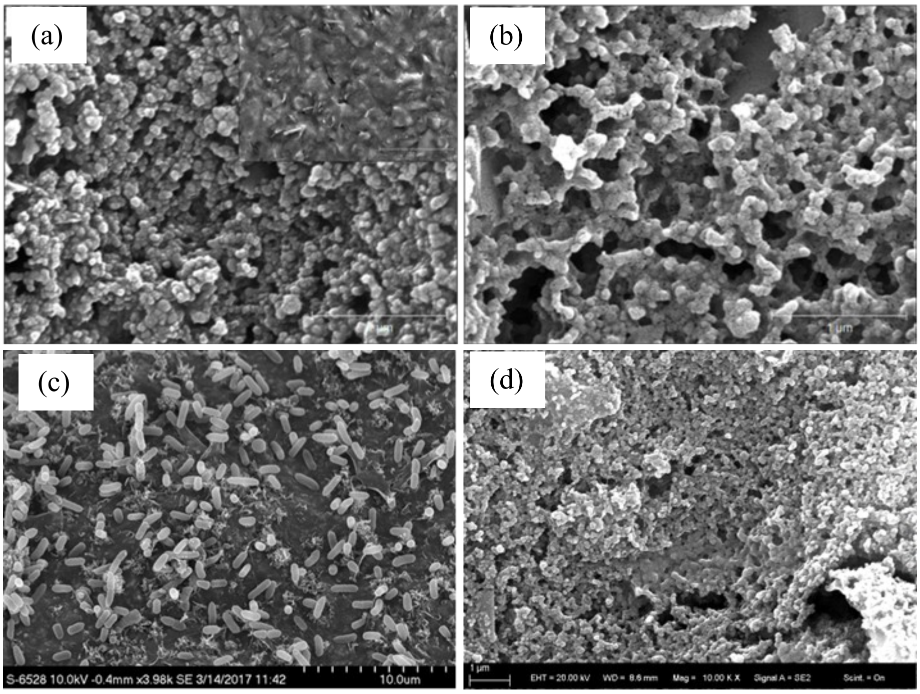

Figure S3. SEM image of the (a) bare SPCE, (b) the β-CD modified SPCE. And images of the electrode when placed in the Pseudomonas fluorescence culture after few days, (c) bare electrode (d) β-CD modified SPCE.

The surface morphologies of the Pβ-CD/SPCE and SPCE were observed by using SEM. (Figure S3a) displays a typical image of the surface of SPCE, which shows a sheet-like structure with a lot of small particles dispersing in between. After the electrodeposition of β-CD polymer, the electrode morphology changed

greatly in (Figure S3b). The surface shows rough and porous structure due to the surface coverage and thickness of cyclodextrin molecules on the electrode surface. The image reveal that the β-CD polymer is well adhered to the electrode surface and covered the entire surface area of the electrode. Bacteria can be clearly observed on the bare electrodes (Figure S3c). However, on CD modified electrodes bacteria are not clearly evident (Figure S3d). Previous publications indicate that CD cavities can cause bacteria to be partially included in the cavities [4]. This could explain why we could not see the bacteria on the surface of the electrode.

RESULT AND DISCUSSION

Electrochemical behaviour of 1-OHPHZ, PCA and PYO (phenazine metabolites) on β-CD /SPCE

The dependence of i_p on ν in (Figure S4a) for anodic and cathodic 1-OHPHZ peaks corresponded to the following equations: $I_{pa}(\mu A) =$ *0.09 ν - 0.33* (R^2= 0.998) and $I_{pc}(\mu A) =$ - 0.09 ν − 0.45 (R^2= 0.998) respectively. These results suggest that the voltametric response in (Figure 1) comes from the surface-confined phenazine molecules, which are included in the cavity of β-CD attached to the surface of the carbon electrode, with a little contribution of the diffusion of the phenazine molecules [5].

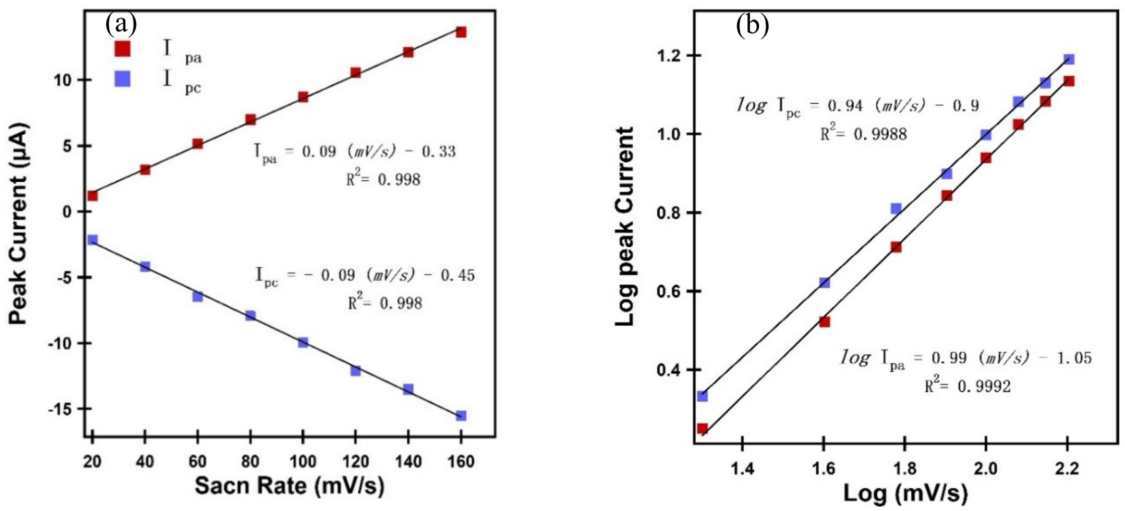

Figure S4. corresponding relationship of (a) I_p vs. ν and (b) $\log I_p$ vs. log ν on β-CD/SPCE for 1-OHPHZ peaks.

The dependence of anodic and cathodic peaks on the scan rate was further evaluated in (Figure S4b), where the logarithm of the phenazine peak current was plotted versus the logarithm of scan rate (at scan rates 20-160mV/s), which confirmed the following equations:

$$\text{Log}\, \nu = 0.99\log\left(i_{pa}\right) - 1.05\ \left(R^2 = 0.9992\right),$$

$$\text{Log}\, \nu = 0.94\log\left(i_{pc}\right) - 0.9\ \left(R^2 = 0.9988\right).$$

For such a relation, the slop value of 1.0 indicate an ideal surface reaction, while the value of 0.5 indicate the diffusion-controlled process [6]. Based on experimental result, the slope value of equations

$\log I_{pa} vs. \log v$ plot was 0.99 which showed that phenazine had strongly adsorbed on the surface of β-CD /SPCE.

Square wave adsorptive stripping voltammetry of phenazine metabolites

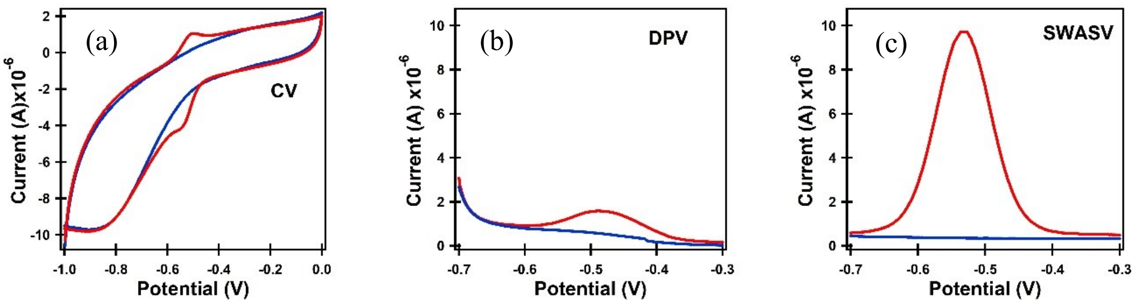

Figure S5: the cyclic voltammogram (a), different pulse voltammogram (b) and square wave adsorptive stripping voltammogram (c) before (blue) and after (red) adding 3 μM 1-OHPHZ into PBS. A pre-accumulation time of 120 s was applied prior to all measurements. As the results shows, SWASV shows peak current 5 times larger than DPV.

Square wave adsorptive stripping voltammetry of phenazine metabolites in pseudomonas species

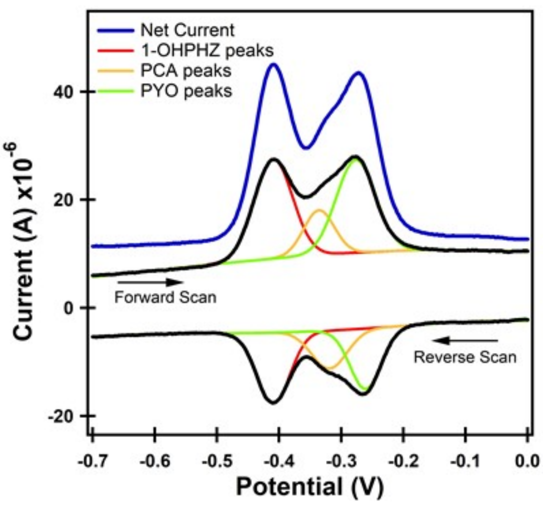

Figure S6: SWASV of the ternary mixture of 12.5 μM 1-OHPHZ, PYO and PCA in PBS, showing forward, backward, and net current (Blue) on β-CD/SPCE. 1-OHPHZ (red peaks), PCA (orange peaks) and PYO (green peaks) in both forward and backward scan. Three peaks corresponding to 1-OHPHZ, PCA and PYO are located at -0.41V, -0.33V, and -0.27V, respectively on the positive and -0.41 V, - 0.31 V, -0.25 V on reverse scan.

Analytical determination of phenazine

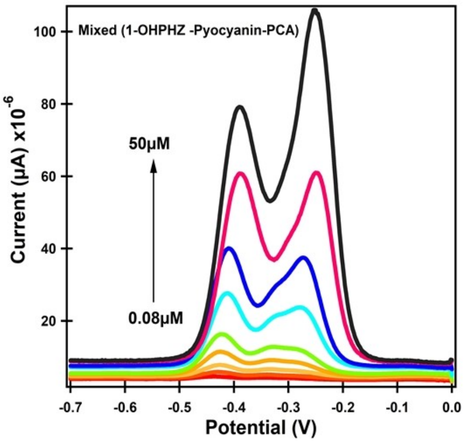

Figure S7: SWAdSV on β-CD modified SPCE for different mixed of phenazine concentration (from 0.08 μM to 50 μM) in PBS buffer.

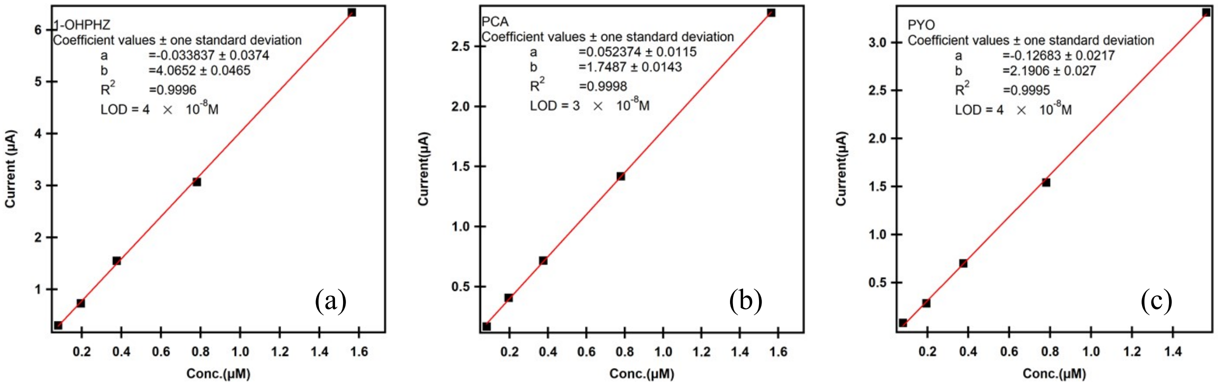

Figure S8. Calculated LOD for (A)1-OHPHZ, (b)PCA and (c)PYO, separately for modified electrode.

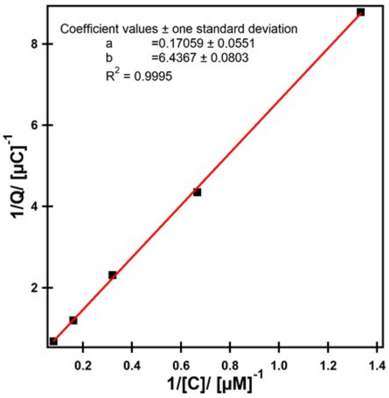

Figure S9: Langmuir isotherm model for 1-OHPHZ on unmodified electrode. From the intercept and slop of 1/ Q versus 1/C plot, Q_{max} = 5.8 µC and k =0.02 μM^{-1}were obtained, respectively.

Electrochemical phenazine detection in Pseudomonas cultures

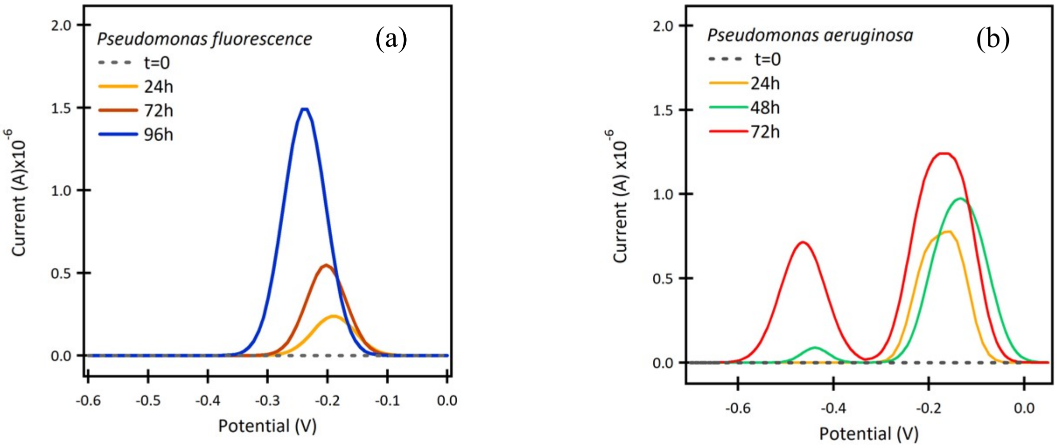

Figure S10. Temporal evolution of SWAdSV responses on β-CD modified electrode in (a) *P. fluorescence*, and (b) *P.aeruginosa* culture.

References

[1] Sidhureddy Boopathi, Tharangattu N. Narayanan and Shanmugam Senthil Kumar, "Improved heterogeneous electron transfer kinetics of fluorinated graphene derivatives," *Nanoscale,* vol. 6, pp. 10140-10146, 2014.

[2] Karolina Sipaa, Mariola Brychta, Andrzej Leniarta, Paweł Urbaniaka, Agnieszka Nosal-Wiercińskab, Bartłomiej Pałeczc, Sławomira Skrzypeka, "β–Cyclodextrins incorporated multi-walled carbon nanotubes modified electrode for the voltammetric determination of the pesticide dichlorophen," *Talanta ,* vol. 176 , p. 625–634, 2018.

[3] Judith F. Rubinson and Yohani P. Kayinamuraa , "Charge transport in conducting polymers: insights from impedance spectroscopy," *Chem. Soc. Rev.,* vol. 38, pp. 3339-3347, 2009.

[4] Zhan, Wenjun, Wei, Ting, Cao, Limin, Hu, Changming, Qu, Yangcui, Yu, Qian and Chen, Hong, "Supramolecular Platform with Switchable Multivalent Affinity: Photo-Reversible Capture and Release of Bacteria," *ACS Applied Materials & Interfaces,* vol. 9, p. 3505–3513, 2017.

[5] Hiroyuki Yamamoto, Yasushi Maeda, and Hiromi Kitano, "Molecular Recognition by Self-Assembled Monolayers of Cyclodextrin on Ag," *J. Phys. Chem. B,* vol. 101, pp. 6855-6860, 1997.

[6] E.Laviron, L.Roullier, "General expression of the linear potential sweep voltammogram for a surface redox reaction with interactions between the adsorbed molecules: Applications to modified electrodes," *Journal of Electroanalytical Chemistry and Interfacial Electrochemistry,* vol. 115, pp. 65-74, 1980.
